# Allograft reconstruction for humeral head defects in the setting of shoulder instability: a systematic review

**DOI:** 10.1016/j.xrrt.2022.07.001

**Published:** 2022-08-07

**Authors:** Allen A. Yazdi, Aseel G. Dib, Joseph W. Elphingstone, Samuel Schick, Brent A. Ponce, Amit M. Momaya, Eugene W. Brabston

**Affiliations:** a University of Alabama at Birmingham Heersink School of Medicine, Birmingham, AL, USA; b Atrium Health Musculoskeletal Institute Department of Orthopaedic Surgery, Charlotte, NC, USA; c University of Alabama at Birmingham Department of Orthopaedic Surgery, Birmingham, AL, USA; d The Hughston Clinic, Columbus, GA, USA

**Keywords:** Hill-Sachs, Reverse Hill-Sachs, Osteochondral allograft, Systematic review, Outcomes, Complications

## Abstract

**Background:**

Glenohumeral joint instability and dislocation are common orthopedic pathologies that can produce osseous humeral head defects such as Hill-Sachs (HS) or Reverse Hill-Sachs (RHS) lesions. Numerous reconstruction techniques have been reported in the literature, including remplissage, disimpaction, and allograft reconstruction. No group has previously assessed the outcomes of allograft reconstruction for RHS lesions, nor compared the outcomes of allograft reconstruction for HS and RHS lesions. In this study, we aim to provide a comprehensive assessment of osteochondral allograft reconstruction for the distinct pathologies of RHS lesions and HS lesions by comparing postreconstruction patient-reported outcomes, complications, and radiographic assessments for each lesion.

**Methods:**

Using Preferred Reporting Items for Systematic Reviews and Meta-analyses guidelines, a systematic review was performed to identify and include studies that reported patient outcomes after the use of osteochondral allografts in the reconstruction of HS or RHS lesions of the humeral head. A comprehensive search of the Google Scholar, PubMed, and Embase databases was conducted with the key terms “allograft,” “Hill-Sachs,” and “reverse Hill-Sachs.”

**Results:**

Eight studies, with a total of 84 patients, were included for review. Of the 84 allograft-treated patients, there were 44 patients with HS lesions and 40 patients with RHS lesions. The average patient age was 27.3 years for HS lesions and 43.0 years for RHS lesions. Postoperative range of motion and average Constant-Murley score (87.9 for HS and 80.1 for RHS) appeared to be greater for those with HS lesions. In addition, 20.5% of HS patients experienced postoperative complications, whereas 42.5% of RHS patients had postoperative complications (*P* = .03). HS and RHS patients experienced similar proportions of graft resorption or collapse rate (22.7% for HS and 12.5% for RHS; *P* = .2).

**Conclusion:**

Patient-reported outcomes indicate that osteochondral allograft reconstruction for large RHS and HS lesions is an acceptable intervention. RHS patients had lower rates of graft resorption and collapse but worse postoperative range of motion and functional outcomes, although these differences were not statistically significant. HS patients experienced significantly fewer complications than those with RHS lesions.

Glenohumeral joint instability and dislocation are common orthopedic pathologies, with a reported incidence of 51 per 100,000 each year.^6^ Regardless of etiology, shoulder dislocations are typically subclassified as anterior, posterior, or inferior based on the position of the displaced humeral head relative to the glenoid. Anterior dislocations are the most common, accounting for 95%-97% of cases, followed by posterior (2%-4%) and inferior (0.5%-1%) dislocations.^1^

Hill-Sachs (HS) or Reverse Hill-Sachs (RHS) lesions are osseous humeral head defects often associated with severe shoulder dislocations. HS lesions are the result of anterior glenohumeral dislocation, whereby the posterosuperolateral humeral head is fractured upon impact with the anterior glenoid. The true incidence of HS lesions is unknown, but they have been reported in 40%-90% of anterior instability events, 65.2% of acute dislocations, and up to 100% of persons with recurrent anterior shoulder instability.^11^^,^^14^ Conversely, RHS lesions are seen following posterior dislocation events, present as osteochondral defects of the anterosuperomedial humeral head, and are reported in up to 86% of posterior shoulder dislocations.^13^

There are numerous surgical techniques to address the humeral head following HS or RHS lesions, including autograft or allograft augmentation, remplissage, disimpaction, and prosthesis replacement. Of the methods used for HS and RHS lesions, allograft reconstruction is often used for the management of large defects with or without glenoid bone injury.^11^ No systematic review exists in the current literature assessing outcomes of allograft reconstruction in RHS lesions. Our study is the first to do so through patient-reported outcomes, complications, and radiographic assessment. In addition, this is the first systematic review to compare the results of allograft reconstruction between HS and RHS lesions. We hypothesized that osteochondral allograft reconstruction will provide similar and satisfactory postoperative outcomes for both HS and RHS lesions.

## Methods

Using Preferred Reporting Items for Systematic Reviews and Meta-analyses guidelines, a systematic review was performed to identify studies that reported patient outcomes after the use of osteochondral allografts in the reconstruction of HS or RHS lesions of the humeral head. A comprehensive search of the Google Scholar, PubMed, and Embase databases was conducted by using different combinations of the key terms “allograft,” “Hill-Sachs,” and “reverse Hill-Sachs,” including (allograft AND Hill-Sachs), (allograft AND reverse Hill-Sachs), (allograft AND Hill-Sachs AND reverse Hill-Sachs), and (allograft AND (Hill-Sachs OR reverse Hill-Sachs)). Our inclusion criteria consisted of level I-IV studies, articles that reported on allograft reconstruction of humeral head defects, and studies that reported clinical and/or radiographic outcomes. Articles were excluded if allograft bone impaction occurred without restoration. Study populations of less than 3 patients, review articles, cadaveric studies, biomechanical studies, and studies not available in English were also excluded.

All duplicate studies were initially removed, and abstracts were reviewed in detail by 2 authors (A.A.Y. and A.G.D.). Disagreements between these authors were arbitrated by the senior author (E.W.B.). Following the initial screening, articles were assessed via full-text review; any disagreements were once again arbitrated by the senior author. The final articles included for review reported patient outcomes after osteochondral allograft reconstruction of HS or RHS lesions of the humeral head.

## Results

### Study selection

**Figure 1:**
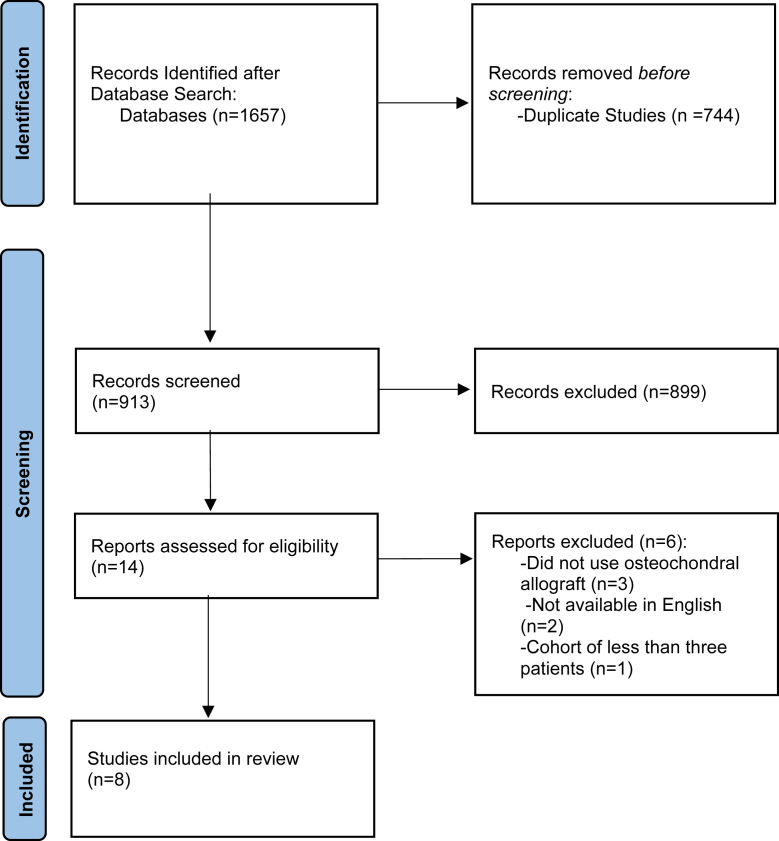
Preferred Reporting Items for Systematic Reviews and Meta-analyses study selection flow diagram.

Initial database searches yielded 1657 records. After duplicates were removed, 913 studies were screened and assessed for eligibility. A total of 8 studies containing 84 patients met inclusion and exclusion criteria in accordance with Preferred Reporting Items for Systematic Reviews and Meta-analyses guidelines (Fig. 1).

### Study characteristics

All 8 studies were case series. Three studies^3^^,^^8^^,^^9^^,^^15^ evaluated 41 patients with HS lesions. Four^2^^,^^4^^,^^5^^,^^7^^,^^10^ studies assessed 38 patients with RHS lesions. One study evaluated 2 RHS patients and 3 patients with humeral head defects due to anterior instability, interpreted by our authors as HS lesions.^12^ In total, our study population was composed of 84 patients, with 44 HS lesions and 40 RHS lesions. The average patient age was 27.3 years for HS lesions and 43.0 years for RHS lesions. The mean follow-up period was 39.5 months for HS lesions and 93.6 months for RHS lesions (Table I).

**Table I:** Study characteristics and patient demographic characteristics.

First author	Year published	Level of evidence	Participant group	Number of patients, shoulders	Lesion type	Graft type	Fixation technique	Concomitant procedures (when indicated)	Mean patient age (y)	Mean follow-up period (mo)	Outcomes
DiPaola^3^	2010	IV	Case series with single treatment group	4, 4	HS	Fresh frozen femoral head (n = 2) or osteochondral allograft plugs (n = 2)	Headless Acutrak screws (femoral head) or Press fit (allograft plugs)	None	33	27.4	ASES score, UCLA score, ROM, radiographs, revision surgery, complications
Miniaci^8^^,^^9^	2018	IV	Case series with single treatment group	18, 18	HS	Irradiated humeral head allograft	Fully threaded cortical screws	Bankart repair and lateral capsulotomy	31.5	50	Constant-Murley score, WOSI score, VAS Pain score, ROM, return to work rate, patient satisfaction, complications, radiographs
Zhuo^15^	2019	IV	Case series with single treatment group	19, 19	HS	Fresh frozen femoral head	Cannulated headless compression screws	Bankart repair, superior labrum repair, and superior labrum debridement	21.7	27.8	ROM, ASES score, Constant-Murley score, Rowe score, patient satisfaction, radiographs, complications
Diklic^2^	2010	IV	Case series with single treatment group	13, 13	RHS	Fresh frozen (n = 12) or Cryopreserved (n = 1) femoral head	Partially threaded cancellous screws	None	42	54	Constant-Murley score, ROM, complications, radiographs
Gerber^4^^,^^5^	2014	IV	Case series with single treatment group	14, 14	RHS	Fresh frozen femoral or humeral head	Press fitted and cancellous lag screws	None	46.96	143.29	Constant-Murley score, age- and gender-adjusted relative Constant-Murley score, SSV, radiographs, revision surgery, complications
Martinez^7^	2013	IV	Case series with single treatment group	6, 6	RHS	Fresh frozen humeral head	Herbert screws	Lateral capsulotomy	31.67	122	Constant-Murley score, ROM, radiographs, revision surgery, return to work, complications
Murphy^10^	2018	IV	Case series with single treatment group	5, 5	RHS	Fresh frozen femoral head	Headless compression screws	Lateral capsulotomy	53.4	34	Constant-Murley score, radiographs, complications
Riff^12^	2017	IV	Case series with single treatment group	5, 5	RHS (n = 2) or HS (n = 3, humeral head osteochondral defects due to anterior stability)	Fresh osteochondral allograft plug (n = 4, with n = 2 for RHS and n = 2 for HS) or Fresh mushroom cap osteochondral allograft (n = 1 for HS)	Press fit or Press fit with supplemental fixation via bioabsorbable compression screws (Bio-Compression; Arthrex) or metallic headless compression screws (Acutrak 2 Standard; Acumed)	Lateral meniscal allograft to resurface glenoid for 1 patient	29.5 (RHS), 29.33 (HS)	66.5	ASES score, VAS Pain score, SST, SF-12P, radiographs, patient satisfaction

*HS*, Hill-Sachs; *RHS*, Reverse Hill-Sachs; *ROM*, range of motion; *SF-12P*, 12-Item Short Form Survey’s Physical Component; *SST*, Simple Shoulder Test; *SSV*, Subjective Shoulder Value; *UCLA*, University of California, Los Angeles; *VAS*, Visual Analog Scale; *WOSI*, Western Ontario Shoulder Instability Index.

### Allograft types used

**Figure 2:**
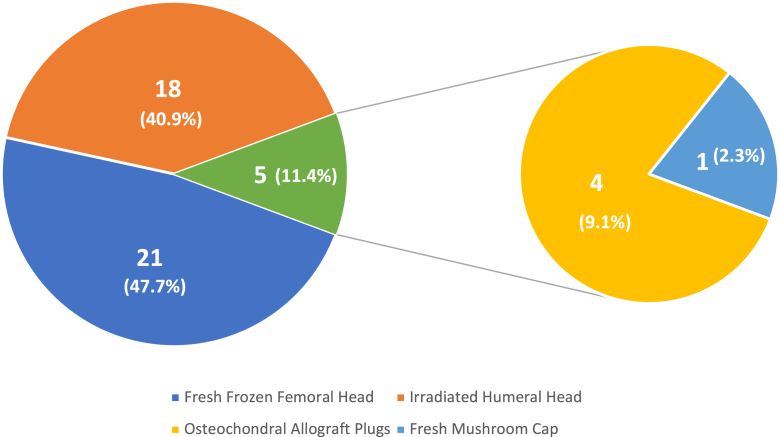
Frequency of allograft type in Hill-Sachs patients.

**Figure 3:**
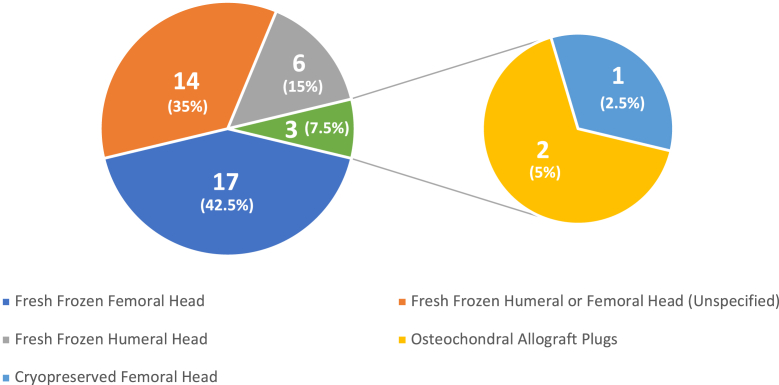
Frequency of allograft type in reverse Hill-Sachs patients. Gerber et al did not specify the number of patients who received fresh frozen humeral head vs. fresh frozen femoral head; thus, it has been placed on the chart as its own portion.

A variety of osteochondral allografts were used for both HS and RHS patients. For HS lesions, fresh frozen femoral head, irradiated humeral head, osteochondral allograft plugs, and fresh mushroom cap osteochondral allografts were used.^3^^,^^8^^,^^9^^,^^12^^,^^15^ Of these, the most used methods were fresh frozen femoral head (21, 47.7%), irradiated humeral head (18, 40.9%), and osteochondral allograft plugs (4, 9.1%; Fig. 2). RHS patients were most frequently treated with fresh frozen femoral head (17, 42.5%), fresh frozen humeral/femoral head (14, 35%), and fresh frozen humeral head (6, 15%) (Fig. 3).^2^^,^^4^^,^^5^^,^^7^^,^^10^^,^^12^

### Range of motion

Range of motion was assessed in 3 of the 4 HS studies (41 total patients). Zhuo et al demonstrated that postoperatively, patients recovered near full range of motion in forward elevation (170° ± 8.2°), external rotation (61.8° ± 8.9°), and internal rotation (T8).^15^ Miniaci et al demonstrated that the average loss of external rotation improved by 30° (from 40° loss preoperatively to just 10° loss postoperatively; Table II).^8^^,^^9^

**Table II:** Hill-Sachs patient outcomes.

First author	Number of patients, shoulders	Graft resorption or collapse rate	Patient satisfaction rate	Range of motion (ROM)	ASES score	Constant-Murley score	Rowe score	Complications	UCLA score	WOSI score	VAS pain score	Return to work rate	Simple Shoulder test (SST)	12-Item short form survey physical component (SF-12P)
DiPaola^3^	4, 4	0%	N/A	Average loss of forward flexion compared with the normal side postoperative: 23° Average loss of external rotation compared with normal side postoperative: 8° Average loss of internal rotation compared with normal side postoperative: 2 levels	Postoperative: 85.3	N/A	N/A	Reflex sympathetic dystrophy (n = 1), Prominent hardware that required removal (n = 1)	Postoperative: 28.4	N/A	N/A	N/A	N/A	N/A
Miniaci^8^^,^^9^	18, 18	11.11%	100%	Average loss of external rotation preoperative: 40° Average loss of external rotation postoperative: 10°	N/A	Postoperative: 87	N/A	Pain in external rotation (n = 2), Osteoarthritis (n = 3), Mild posterior subluxation (n = 1)	N/A	Preoperative: 1882 Postoperative: 381	Preoperative: 72.5 Postoperative: 22.5	89%	N/A	N/A
Riff^12^	3,3	0%	66.67%	N/A	Postoperative: 79	N/A	N/A	None	N/A	N/A	Postoperative: 1.6	N/A	Postoperative: 80	Postoperative: 50
Zhuo^15^	19, 19	43.10%	94.70%	Forward elevation-Preoperative: 160.3 ± 7.72° Postoperative: 170.0 ± 8.16° External rotation-Preoperative: 54.7 ± 6.73° Postoperative: 61.8 ± 8.85° Internal rotation-Preoperative: T9 Postoperative: T8	Preoperative: 53.2 ± 6.83 Postoperative: 96.9 ± 2.43	Preoperative: 81.1 ± 5.11 Postoperative: 88.8 ± 3.48	Preoperative: 23.6 ± 7.22 Postoperative: 97.6 ± 2.12	Pain in the operative shoulder (n = 1)	N/A	N/A	N/A	N/A	N/A	N/A

*N/A*, not available; *UCLA*, University of California, Los Angeles; *VAS*, Visual Analog Scale; *WOSI*, Western Ontario Shoulder Instability Index.

Two of the 5 RHS studies (19 total patients) evaluated postoperative range of motion. Martinez et al reported that postoperatively, patients had average range of motion values of 116.7° for forward elevation, 115.8° for lateral elevation, 69.2° for external rotation, and 69.2° for internal rotation.^7^ Meanwhile, Diklic et al. reported an average postoperative Constant-Murley range of movement subscore of 36.2/40 for their patients (Table III).^2^

**Table III:** Reverse Hill-Sachs patient outcomes.

First author	Number of patients, shoulders	Graft resorption or collapse rate	Patient satisfaction rate	Range of motion (ROM)	ASES score	Constant-Murley score	Complications	Return to work average (weeks)	VAS pain score	Subjective Shoulder Value (SSV)	Simple shoulder test (SST)	12-Item short form survey physical component (SF-12P)
Diklic^2^	13, 13	7.69%	N/A	Constant-Murley range of movement subscore postoperative: 36.2	N/A	Postoperative: 86.8 Mean postoperative pain subscore: 12.7 Mean activities of daily living subscore: 17.2 Mean range of movement subscore: 36.2 Mean strength subscore: 20.5	Spontaneous osteonecrosis of the humeral head (n = 1), occasional mild night pain without the need for analgesia (n = 3), Moderate slight pain that required the use of oral analgesics (n = 1)	N/A	N/A	N/A	N/A	N/A
Gerber^4^^,^^5^	14, 14	7.14%	N/A	N/A	N/A	Postoperative: 77.5 Age- and gender-adjusted relative postop Constant Murley score: 89.5%	Osteoarthritis (n = 7), prosthetic revision (n = 2), static posterior subluxation (n = 1), diffuse osteochondromatosis (n = 1), secondary avascular necrosis of the humeral head (n = 1)	N/A	N/A	83.90%	N/A	N/A
Martinez^7^	6, 6	33.33%	N/A	Forward elevation postoperative: 116.67° Lateral elevation postoperative: 115.83° External rotation postoperative: 69.167° Internal rotation postoperative: 69.167°	N/A	Postoperative: 69.167	Pain, clicking, catching and stiffness (n = 3), shoulder osteoarthrosis (n = 3), revision shoulder arthroplasty (n = 3)	16	N/A	N/A	N/A	N/A
Murphy^10^	5, 5	20%	N/A	N/A	N/A	Postoperative: 83	Partial flattening of the articular surface of the graft (n = 1), articular retraction of the graft (n = 1)	N/A	N/A	N/A	N/A	N/A
Riff^12^	2,2	0%	100%	N/A	Postoperative: 79	N/A	None	N/A	Postoperative: 1.6	N/A	Postoperative: 80	Postoperative: 50

*N/A*, not available; *VAS*, Visual Analog Scale.

### Functional outcomes

Several functional outcome surveys were evaluated in the collected studies, including the American Shoulder and Elbow (ASES) score; Constant-Murley score; Rowe score; University of California, Los Angeles score; Western Ontario Shoulder Instability Index score; Visual Analog Scale (VAS) pain score; Subjective Shoulder Value; Simple Shoulder Test score; and the 12-Item Short Form Survey’s Physical Component. Only the ASES, Constant-Murley, and VAS pain scores were reported in more than one HS study (Table II). Meanwhile, only the Constant-Murley score was reported in more than one RHS study (Table III).

Postoperative Constant-Murley score was assessed in 2 of the 4 HS studies (37 total patients), with an average score of 87.9.^8^^,^^9^^,^^15^ Four of the 5 RHS studies (38 total patients) reported Constant-Murley scores, with an average score of 80.1.^2^^,^^4^^,^^5^^,^^7^^,^^10^ Postoperative ASES score was reported in 3 of the 4 HS studies (26 total patients), with an average score of 93.1.^3^^,^^12^^,^^15^ Only one RHS study (2 total patients) reported ASES score, with an average score of 79.^12^ VAS pain score was reported in 2 of the 4 HS studies (21 total patients), with improvements in average shoulder pain score after operation in both studies.^8^^,^^9^^,^^12^ Only one RHS study (2 patients) reported average improvement in VAS shoulder pain score after operation.^12^

### Radiographic outcomes

Across all 4 HS studies, the average graft resorption or collapse rate was 22.7% (10/44 total patients; Table II).^3^^,^^8^^,^^9^^,^^12^^,^^15^ Meanwhile, graft resorption or collapse rate was reported in all 5 RHS studies, with the average rate being 12.5% (5/40 total patients; Table III).^2^^,^^4^^,^^5^^,^^7^^,^^10^^,^^12^ There was no statistically significant difference in terms of graft resorption or collapse rate (*P* = .22). The odds ratio for HS relative to RHS was 2.059 (95% confidence interval, 0.637-6.651).

### Patient satisfaction

Patient satisfaction was reported in 3 HS studies (40 total patients), with an average satisfaction rate of 95% (Table II).^8^^,^^9^^,^^12^^,^^15^ Patient satisfaction rate was only provided in one RHS study (2 total patients) and resulted in an average rate of 100% (Table III).^12^

### Complications

Of the 44 HS patients, 9 suffered postoperative complications: pain (n = 3), osteoarthritis (n = 3), mild posterior subluxation (n = 1), reflex sympathetic dystrophy (n = 1), and prominent hardware that required removal (n = 1). In total, the weighted mean incidence of postoperative complications for HS patients was 20.5%. Of those who suffered complications, the number of complications per patient was 1 (Table II).^3^^,^^8^^,^^9^^,^^12^^,^^15^

Of the 40 RHS patients, 17 suffered postoperative complications: shoulder pain (n = 7), osteoarthritis (n = 7), revision arthroplasty (n = 5), osteoarthrosis (n = 3), clicking (n = 3), catching (n = 3), stiffness (n = 3), osteonecrosis (n = 2), partial flattening of the graft (n = 1), retraction of the graft (n = 1), static posterior subluxation (n = 1), and diffuse osteochondromatosis (n = 1). In total, the weighted mean incidence of postoperative complications for RHS patients was 42.5%. Of those who suffered complications, the average number of complications per patient was 2.2 (Table III).^2^^,^^4^^,^^5^^,^^7^^,^^10^^,^^12^

RHS and HS had significantly different postoperative complication rates (*P* = .029). The odds ratio for HS relative to RHS was 2.874 (95% confidence interval, 1.096-7538).

## Discussion

To our knowledge, this is the first study to provide a comprehensive assessment of allograft reconstruction for RHS lesions through patient-reported outcomes, complications, and radiographic assessment. By extension, this study is also the first to compare the results of allograft reconstruction between HS and RHS lesions. Following our review, we found that osteochondral allograft reconstruction for HS and RHS lesions provides similar and satisfactory outcomes. When comparing the 2 patient populations, HS patients appeared to have greater average functional scores and postoperative range of motion. In addition, postoperative complication rates for HS patients were found to be significantly lower compared with RHS patients. Although it appears that relatively fewer RHS patients reported graft resorption or collapse, there was no statistically significant difference found between the 2 groups.

For both functional scores and postoperative range of motion, HS patients appeared to experience better outcomes than their RHS counterparts (Tables II and III). However, after further examination, we believe these values are potentially skewed by the differences in average patient age (27.3 years for HS and 43.0 for RHS) and mean follow-up period (39.5 months for HS and 93.6 months for RHS) seen in Table I. A strong argument supporting this claim can be seen in Martinez et al and Gerber et al, where the mean follow-up periods (122 and 143.3 months respectively) were the longest among all HS and RHS studies. Compared with the rest of the studies, these 2 RHS studies reported the lowest functional scores and postoperative range of motion values, while also reporting the highest rates of complications, another potential explanation for the worse outcomes.^4^^,^^5^^,^^7^

As for the significant difference in postoperative complication rates, 20.5% of HS patients, and 42.5% of RHS patients reported complications.^2^, ^3^, ^4^, ^5^^,^^7^, ^8^, ^9^, ^10^^,^^12^^,^^15^ Once again, we believe this stark difference is potentially due to the older average age and longer mean follow-up period for RHS patients. With an older population and a longer follow-up interval, RHS patients had more time to potentially develop postoperative complications, especially those that develop via arthritic processes. This is corroborated by the fact that most RHS complications were reported by Martinez et al and Gerber et al, despite these studies comprising less than half of the RHS study patient population.^2^^,^^4^^,^^5^^,^^7^^,^^10^^,^^12^

Although HS patients fared significantly better in terms of complications, there was no statistically significant difference found between HS and RHS patients in terms of graft resorption or collapse rate.^2^, ^3^, ^4^, ^5^^,^^7^, ^8^, ^9^, ^10^^,^^12^^,^^15^ Nonetheless, clinical significance of these findings should not be undervalued, as nearly one-fourth of HS patients and one-eighth of RHS patients experienced graft resorption or collapse. In addition, Zhuo et al reported significant differences between their resorption and nonresorption groups in terms of age, duration of instability, and preoperative size of HS lesion.^15^ Taking these findings into account, it is critical to preoperatively evaluate HS patients on an individual basis to ensure chances of graft resorption or collapse remain low. As for RHS patients, more research is needed to be done to determine if differences are found between resorption and nonresorption groups.

As with every surgical intervention, allograft reconstruction for an HS or RHS lesion has its advantages and disadvantages. To date, prior literature supports the use of allografts as a powerful tool for reconstruction of the humeral head. Benefits such as high functional outcome scores and improvements in range of motion must be weighed against the risks of postoperative complications and graft resorption or collapse. Our findings can be of great value, as they provide clinicians an up-to-date summary of allograft reconstruction for both disease processes. However, further work is needed to assess the optimal allograft type for humeral head reconstruction.

### Limitations

Our study has several limitations. With limited published literature on allograft reconstruction for RHS lesions, the primary limitation of our study is small patient populations. Demographic differences between RHS and HS groups, as well as differences in the underlying pathology, limit our study conclusions. In addition, variation in the individual studies, including surgical techniques, follow-up periods, and allograft types, should not be overlooked. It is also difficult to directly compare the outcomes of the studies, as few patient-reported outcomes or range of motion measures were consistent between studies. Finally, as most studies lacked preoperative assessment values, we could not generate effect sizes for meta-analysis.

## Conclusion

In this study, we demonstrated that humeral head defects reconstructed with osteochondral allograft produce similar and satisfactory outcomes for large HS and RHS lesions. RHS patients had lower rates of graft resorption and collapse but worse postoperative range of motion and functional outcomes, although these differences were not statistically significant. HS patients experienced significantly fewer complications than those with RHS lesions. Given the limited overlap in reported outcomes between studies and the paucity of published cases of HS and RHS allograft reconstruction, more studies are needed to better characterize patient populations and predict patient outcomes.

## Disclaimers

Funding: No funding was disclosed by the authors.

Conflicts of interest: The authors, their immediate families, and any research foundation with which they are affiliated have not received any financial payments or other benefits from any commercial entity related to the subject of this article.

## References

[1] Brady W.J., Knuth C.J., Pirrallo R.G. (1995). Bilateral inferior glenohumeral dislocation: luxatio erecta, an unusual presentation of a rare disorder. J Emerg Med.

[2] Diklic I.D., Ganic Z.D., Blagojevic Z.D., Nho S.J., Romeo A.A. (2010). Treatment of locked chronic posterior dislocation of the shoulder by reconstruction of the defect in the humeral head with an allograft. J Bone Joint Surg Br.

[3] DiPaola M.J., Jazrawi L.M., Rokito A.S., Kwon Y.W., Patel L., Pahk B.Z.J. (2010). Management of humeral and glenoid bone loss--associated with glenohumeral instability. Bull NYU Hosp Jt Dis.

[4] Gerber C., Catanzaro S., Jundt-Ecker M., Farshad M. (2014). Long-term outcome of segmental reconstruction of the humeral head for the treatment of locked posterior dislocation of the shoulder. J Shoulder Elbow Surg.

[5] Gerber C., Lambert S.M. (1996). Allograft reconstruction of segmental defects of the humeral head for the treatment of chronic locked posterior dislocation of the shoulder. J Bone Joint Surg Am.

[6] Hindle P., Davidson E.K., Biant L.C., Court-Brown C.M. (2013). Appendicular joint dislocations. Injury.

[7] Martinez A.A., Navarro E., Iglesias D., Domingo J., Calvo A., Carbonel I. (2013). Long-term follow-up of allograft reconstruction of segmental defects of the humeral head associated with posterior dislocation of the shoulder. Injury.

[8] Miniaci A., Berlet G., Hand C., Lin A. (2018). Segmental humeral head allografts for recurrent anterior instability of the shoulder with large Hill-Sachs defects: a two to 8 year follow up. Orthop Proc.

[9] Miniaci A., Gish M.W. (2004). Management of anterior glenohumeral instability associated with large Hill–Sachs defects. Tech Shoulder Elbow Surg.

[10] Murphy L.E., Tucker A., Charlwood A.P. (2018). Fresh frozen femoral head osteochondral allograft reconstruction of the humeral head reverse Hill Sachs lesion. J Orthop.

[11] Provencher M.T., Frank R.M., LeClere L.E., Metzger P.D., Ryu J.J., Bernhardson L.T.A. (2012). The Hill-Sachs lesion: diagnosis, classification, and management. J Am Acad Orthop Surg.

[12] Riff A.J., Yanke A.B., Shin J.J., Romeo A.A., Cole B.J. (2017). Midterm results of osteochondral allograft transplantation to the humeral head. J Shoulder Elbow Surg.

[13] Saupe N., White L.M., Bleakney R., Schweitzer M.E., Recht M.P., Jost B. (2008). Acute traumatic posterior shoulder dislocation: MR findings. Radiology.

[14] Yiannakopoulos C.K., Mataragas E., Antonogiannakis E. (2007). A comparison of the spectrum of intra-articular lesions in acute and chronic anterior shoulder instability. Arthroscopy.

[15] Zhuo H., Xu Y., Zhu F., Pan L., Li J. (2019). Osteochondral allograft transplantation for large Hill-Sachs lesions: a retrospective case series with a minimum 2-year follow-up. J Orthop Surg Res.

